# A Population-Based Clinical Trial of Irinotecan and Carboplatin

**DOI:** 10.1155/2009/458528

**Published:** 2009-04-23

**Authors:** Derick Lau, Minh Huynh, Jewel Johl

**Affiliations:** ^1^Davis Cancer Center, University of California, Sacramento, CA 95817, USA; ^2^Department of Veterans Affairs Northern California Health Care System (VANCHCS), University of California, Sacramento, CA 95817, USA

## Abstract

*Purpose*. Phase I trials of anticancer drugs are commonly conducted using the method of modified Fibonacci. We have developed a population-based design for phase I trials of combining anticancer drugs such as irinotecan and carboplatin. *Patients and Methods*. Intrapatient dose escalation of irinotecan and carboplatin was performed according to a predetermined schema to reach individual dose-limiting toxicity (DLT) in 50 patients with solid tumors refractory to previous chemotherapy. The individual toxicity-limiting dose levels were analyzed for normal distribution using the method of Ryan-Joiner and subsequently used to determine a population-based maximum tolerated dose (pMTD). For comparison, a simulation study was performed using the method of modified Fibonacci. *Results*. The most common dose-limiting toxicities (DLTs) included neutropenia (58%), thrombocytopenia (16%), and diarrhea (8%). The frequency of individual toxicity-limiting dose levels of 50 patients approximated a normal distribution. The dose levels associated with individual limiting toxicities ranged from level 1 (irinotecan 100 mg/m^2^ and carboplatin AUC = 4 mg/mL x min) to level 8 (irinotecan 350 mg/m^2^ and carboplatin AUC = 6). The pMTD was determined to be dose level 3 (150 mg/m^2^ for irinotecan and AUC = 5 for carboplatin). In contrast, the MTD was determined to be dose level 4 (200 mg/m^2^ for irinotecan and AUC 5 for carboplatin) by modified-Fibonacci simulation. *Conclusions*. The population-based design of phase I trial allows optimization of dose intensity and derivation of a pMTD. The pMTD has been applied in phase II trial of irinotecan and carboplatin in patients with small-cell lung cancer.

## 1. Introduction

Phase I clinical trials of new anticancer agents have been commonly conducted using the method of modified Fibonacci [1]. In brief, 3 patients are treated at a starting dose which is typically one tenth of the dose that is lethal to 10% of animals defined in preclinical studies. If none of the 3 patients experiences DLT, then the next 3 patients will be treated at the next higher dose. If DLT is observed, additional patients will be treated at the same or lower dose level to determine MTD according to a predetermined schema. The MTD is defined as the highest dose reached for which the incidence of DLT occurs in less than 33% of the subjects. Typically, intrapatient dose escalation is not allowed.

There are several shortcomings associated with the modified-Fibonacci design. It has long been recognized that a substantial number of patients are likely to be treated at subtherapeutic doses [2, 3]. This is particularly true for drugs with potential anticancer activity. Since the primary purpose of phase I trials is to determine DLT and MTD, the efficacy of the drug may not be evident for certain tumor types as there are only a small number of patients enrolled into the trial. Furthermore, the modified-Fibonacci design does not take into account individual variations in therapeutic and toxicologic responses due to genomic polymorphisms [4, 5]. In addition, since there is a limit of 3 subjects allowed for each cohort, a waiting period of up to four weeks is commonly required before enrollment of the next cohort of subjects. This latter requirement creates anxiety of waiting for eligible patients.

Several alternative phase I designs have been proposed which limit the number of patients accrued at each dose level and accelerate the dose escalation process [1, 6]. There has also been an increase in the number of clinical trials that include a component of intrapatient dose escalation although no formal validation with the modified-Fibonacci approach has been reported [7–9]. We have pioneered a population-based design to maximize therapeutic efficacy, to provide preliminary efficacy information and to allow derivation of a pMTD for subsequent phase II trials.

Irinotecan and cisplatin have been shown to have promising efficacy in patients with small-cell lung cancer in phase III trial where irinotecan was given on a weekly schedule [10]. We wished to perform a population-based phase I trial of irinotecan, given every 3 weeks, and carboplatin, a platinated anticancer drug which is generally better tolerated than its cisplatin analog.

## 2. Methods

### 2.1. Patients and Intrapatient Dose Escalation

Eligibility criteria included patients with a diagnosis of advanced solid tumor not curable by standard therapies; measurable disease; Zubrod performance status of 0–2; age >18 years; prior chemotherapy was allowed except irinotecan or carboplatin; adequate hematologic (ANC >1500 and platelets >100 000/mL), hepatic (total bilirubin and SGOT <2× upper limit of normal), and renal (serum creatinine <1.5 mg/dl) functions. All patients signed informed consent in accordance with guidelines of the institutional review board.

Intrapatient dose-escalation schema is shown in Table 1. This schema was extrapolated from the results of a previous population-based phase I trial of irinotecan and epirubicin in patients with solid tumors, in which the pMTD was determined to be 100 mg/m^2^ for irinotecan and 50 mg/m^2^ for epirubicin in previously chemotherapy-treated patients [11]. All patients were started at dose level 3, which consisted of irinotecan, 150 mg/m^2^, infused intravenously over 90 minutes, and carboplatin, AUC 5 mg/mL × min, given as an intravenous bolus on day 1. The carboplatin dose was determined according to the Calvert formula [12] and a calculated creatinine clearance [13]. Toxicity was graded according to the common toxicity criteria, Version 2, of the National Cancer Institute (NCI). Treatment was repeated every 21 days and the dose for subsequent cycle was escalated by one dose level as outlined in Table 1 if no grade III or IV toxicity was observed. For any grade IV neutropenia, grade III or IV thrombocytopenia, or grade III or IV nonhematologic toxicity, the dose was decreased by one level as outlined in Table 1. No prophylactic growth factor was allowed. Patients remained on treatment for at least 6 cycles, or until there was evidence of disease progression, intolerable toxicity, or voluntary withdrawal.

In accordance to common practice, DLT was defined as grade III or IV thrombocytopenia, grade IV neutropenia, or any grade III or IV nonhematologic toxicity. Similar to the modified-Fibonacci method [1], the pMTD was defined as the highest dose level that caused DLT in less than 33% of the population studied (DLT_33%_). For each subject who did not reach DLT, the dose level associated with DLT was assumed to be one level higher than the last one the subject received prior to coming off the study.

Responses were evaluated after 3 cycles of treatment. Tumor measurement was performed according to the NCI RECIST criteria [14].

### 2.2. Statistical Analysis

Based on the results of a previous population-based phase I trial of irinotecan and epirubicin, the optimal number of subjects was estimated to be 25 to 50 for reaching a Gaussian or normal distribution of individual toxicity-limiting dose levels [11]. The computer program of Ryan-Joiner test (Minitab Release 14, statistical software for Windows, Minitab Inc., State College, Pa, USA) was employed to determine the normality of the distribution of individual toxicity-limiting dose levels [15]. In the Ryan-Joiner analysis, a correlation coefficient, *r*, was obtained to determine the degree of normality. The more closer was *r* to 1, the more normal was the distribution

(1) $$r=\frac{\sum Y_{i}b_{i}}{\sqrt{s^{2}(n-1)\sum b_{i}^{2}}},$$

where *Y*_*i*_ was an individual toxicity-limiting dose level, *b*_*i*_ was the probability percentage point of the normal distribution associated with the individual dose level, *s* denoted the sample variance, and *n* was the sample size.

To determine the pMTD, the mean (M_DLT_) and standard deviation (SD_DLT_) of the 50 individual dose levels associated with DLT were calculated. The pMTD was the estimated highest dose level that caused DLT in less than 33% of the subjects (DLT_33%_). Assuming that the individual DLT followed a normal distribution, the 33% cutoff point was just below 17% to the left from the mean. Therefore,

(2) $${\text{DLT}}_{33\%}={\text{M}}_{\text{DLT}}-({\text{SD}}_{\text{DLT}}\times Z_{17\%}),$$

where *Z*_17%_ is the level of standard deviation to the left of the M_DLT_ with a probability of 17% under a normal distribution curve [16]. Thus, the pMTD would be one dose level lower than DLT_33%_.

### 2.3. Modified-Fibonacci Simulation

To compare the pMTD derived from the population-based approach with the MTD that otherwise would have been obtained with the modified-Fibonacci method, a simulation exercise was performed using the data from the current study. The Fibonacci simulation would also allow us to compare the number of subjects required using each approach. The procedures for simulation were performed according to the 3 + 3 rule of modified Fibonacci [1] with the following assumptions. (1) The first cohort of subjects started at dose level 3; (2) if a patient treated at a particular dose level had not experienced DLT, it was assumed that no DLT would have been experienced at a lower starting dose; (3) if a patient had experienced DLT at a particular dose level, it was assumed that this patient would have experienced DLT at a higher dose level; (4) the MTD is defined as the highest dose reached for which the incidence of DLT is less than 33% in the subjects [1].

## 3. Results

### 3.1. Patient Characteristics

A total of 50 patients were enrolled from a single institution within a period of 24 months. Patient characteristics are summarized in Table 2. There were 27 male and 23 female patients. The median age was 61 years with a range of 35–83 years. Forty-seven patients (94%) had a Zubrod performance status of 0 or 1. All the patients had previously received at least one regimen of chemotherapy and the mean number of previous chemotherapy regimens was 1.6 (range 1 to 3). Forty-two percent of the patients had a diagnosis of nonsmall-cell lung cancer, 20% with small-cell lung cancer, 20% with gastrointestinal cancer, 6% with head/neck cancer, and 12% with a variety of solid cancers.

### 3.2. Derivation of Population-Based MTD

Among the 50 patients enrolled, the median number of cycles of irinotecan and carboplatin, that had been delivered, was three. Treatment was discontinued before reaching DLT in 10 patients due to disease progression or voluntary withdrawal. Dose escalation to DLT was achieved in the remaining 40 patients. As shown in Table 3, the most common grade III/IV toxicities or dose-limiting toxicities were neutropenia in 58%, thrombocytopenia in 16%, diarrhea in 8%, nausea/emesis in 8%, and asthenia in 4% of the patients.

**Figure 1:**
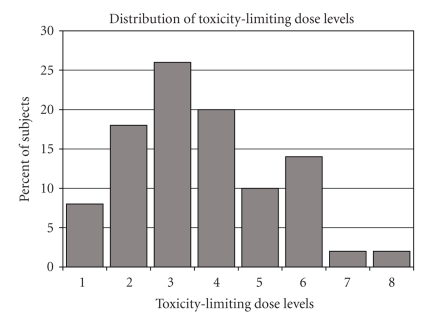
Distribution of individual dose levels associated with dose-limiting toxicity (DLT) of irinotecan and carboplatin (*N* = 50).

**Figure 2:**
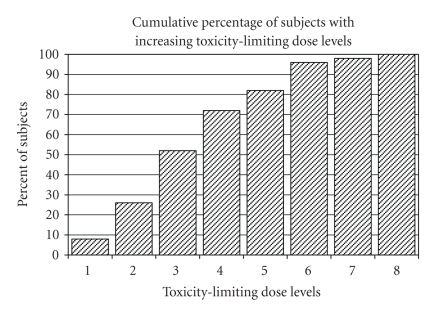
Cumulative percent of patients experienced dose-limiting toxicity (DLT) with increasing dose levels of irinotecan and carboplatin (*N* = 50).

The distribution of individual toxicity-limiting dose levels ranged from level 1 (100 mg/m^2^ of irinotecan and AUC 4 of carboplatin) to level 8 (irinotecan 350 mg/m^2^ and carboplatin AUC 6) as shown in Figure 1. The individual toxicity-limiting dose levels approximated a normal distribution with a correlation coefficient, *r*, of 0.992, based on the Ryan-Joiner analysis. The cumulative percentage of subjects with increasing toxicity-limiting dose levels are shown in Figure 2. Assuming a normal distribution of the toxicity-limiting dose levels, the DLT_33%_ was calculated to be 3.9. By the definition that pMTD was one dose level lower than that of DLT_33%_, the pMTD was thus 2.9 which approximated 150 mg/m^2^ of irinotecan and AUC 5 of carboplatin.

Forty of the 50 patients were assessable for responses. Ten patients were not assessable for response due to early disease progression or withdrawal from the study before having received 3 cycles of treatment. The best responses by tumor type are shown in Table 4. Of note, the response rate was 40% for small-cell lung cancer, 24% for nonsmall-cell lung cancer, and 30% for gastrointestinal malignancies consisting mainly of esophageal and gastric cancers. There was no apparent correlation between response rate and individual MTD levels.

### 3.3. Modified-Fibonacci Simulation

By the Fibonacci simulation, an MTD of dose level 4 (200 mg/m^2^ of irinotecan and AUC 5 of carboplatin) was derived after simulation of 18 consecutive patients.

Table 5 outlines the features of the population-based approach versus the modified-Fibonacci method.

## 4. Discussion

In this study, we have demonstrated the feasibility of applying a population-based approach in conducting phase I trial and the derivation of a pMTD for irinotecan and carboplatin for subsequent phase II trials.

This study clearly illustrated the phenomenon of population polymorphism in clinical practice. We showed that the individual DLTs occurred over a range of 8 dose levels. At dose level 1, the dose for irinotecan was 100 mg/m^2^ and carboplatin was AUC of 4. At dose level 8, the corresponding doses were 350 mg/m^2^ and AUC of 6, respectively. It is quite remarkable to see that one patient could tolerate 3.5 times higher of irinotecan and 1.5 times higher of carboplatin than another patient. This marked degree of interpatient dose variation was most likely due to interpatient variability in pharmacogenomics. In this regard, irinotecan is a typical example with individual variability in pharmacokinetics and toxicity. Following intravenous administration, irinotecan is converted to an active metabolite, SN-38, which is subsequently deactivated by uridine diphosphate glucuronosyltransferase isoform 1A1 (UGT1A1). It has been demonstrated that variability in pharmacokinetics and toxicity of irinotecan correlates with polymorphisms of the UGT1A1 promoter [17, 18]. In this study, we did not monitor the pharmacokinetics of irinotecan or pharmacogenetics of UGT1A1. Nevertheless, we believe that the intrapatient dose-escalation scheme is appropriate for conducting phase I trials with a drug such as irinotecan.

Maximum tolerated doses of carboplatin and irinotecan have been reported in previous phase I trials although irinotecan typically was administered on a weekly schedule in these studies. Based on phase I trial on patients with relapsed or refractory advanced malignancies as reported by Jones et al., the MTD was irinotecan at 60 mg/m^2^ on days 1 and 8 in combination with carboplatin at AUC 4.0 on day 1 for 28-day cycles [19]. In another phase 1 trial on subjects with ovarian cancer previously treated with cisplatin-based chemotherapy, Yonemori et al. reported that the MTD of the irinotecan/carboplatin combination was 60 mg/m^2^ on days 1, 8, and 15, and 5 mg mL/minute on day 1, respectively, for 28-day cycles [20]. Undoubtedly, the delivery of the 3-week regimen as reported in our study is more convenient and presumably less costly.

In phase I trials employing the modified-Fibonacci design, the initial cohorts of subjects are commonly given subtherapeutic doses of an anticancer drug. On the other hand, some subjects in the subsequent cohorts may receive toxic doses as dose escalation is based on the 3 + 3 rule [1]. In the intrapatient dose-escalation design, each subject is started on a low dose and is entitled to receive a higher dose as long as there is no DLT. Thus, this latter approach tends to minimize toxicity and maximize efficacy in each subject.

In traditional phase I trials, only 3 subjects can be enrolled at a dose level at one time. Generally, they have to be observed for a month before the next cohort of subjects can be enrolled at the next dose level. This requirement results in unnecessary waiting for potentially eligible patients and may prolong the conduct of a study. With the population-based method, subjects commonly can be enrolled continuously to a study without a mandatory waiting period.

There are potential disadvantages associated with the population-based design. A patient may develop progressive disease or leave the study for other reasons before subsequent dose escalation and determination of DLT can be achieved. Dose escalation in the same patient may result in cumulative toxicity and, theoretically, an MTD level lower than that obtained with the Fibonacci method. However, in our study, the DLTs were reached with the 3 lowest dose levels in more than 50% of subjects. In addition, there were subjects who tolerated more than 4 sequentially escalated doses without experiencing obvious cumulative toxicity. These observations indicate that the intolerance to treatment is intrinsic to each subject rather than due to cumulative toxicity.

With a population-based approach, it is apparent that a larger number of subjects are required than that for the modified-Fibonacci design. In our study, we had enrolled 50 subjects to derive a pMTD. In comparison, only 18 subjects would have been needed with the Fibonacci simulation. With a limitation of the number of subjects, it may yield a MTD not representative of a population. Based on the results of Ryan-Joiner analysis and pMTDs, we estimated that 40 patients would have been adequate to obtain similar results with the population-based approach. With a larger number of patients having a variety of tumor types, however, it provides a better opportunity to evaluate efficacy in a certain tumor type. For example, in our study, we observed partial response in 4 of 10 patients with relapsed small-cell lung cancer. This observation has led to phase II trial which employed the pMTD of irinotecan (150 mg/m^2^) and carboplatin (AUC = 5) derived in this study in patients with relapsed small-cell lung cancer [21].

The population-based design is open ended in regard to the number of patients to be accrued and dose levels to be escalated. This design appears most appropriate for a combination of anticancer drugs with proven single-agent anticancer activity and toxicity. It may not be appropriate for anticancer drugs with low toxicity profiles such as the molecular-targeting agents.

We conclude that the population-based design is a feasible approach for conducting phase I trials of anticancer drugs. This approach allows derivation of a pMTD without causing obvious cumulative toxicity.

## Figures and Tables

**Table 1:** Schema of dose levels for irinotecan and carboplatin.

Dose level	Irinotecan (mg/m^2^)	Carboplatin AUC
1	100	4
2	100	5
3	150	5
4	200	5
5	250	5
6	250	6
7	300	6
8	350	6

**Table 2:** Patient characteristics.

Number of patients	50
Male/female	27/23

Age (yr)	
* *range	35–83
* *Median	61

Performance status	
* *0	12
* *1	35
* *2	3

Number of previous chemotherapies	
* *1	25
* *2	20
* *3	5

Tumor types	
* *Nonsmall-cell lung	21
* *Small-cell lung	10
* *Gastrointestinal	10
* *Head/neck	3
* *Miscellaneous	6

**Table 3:** Dose-limiting grade III/IV toxicities (*N* = 50).

Toxicities	Number of patients (%)
Neutropenia	29 (58)
Thrombocytopenia	8 (16)
Diarrhea	4 (8)
Nausea/emesis	4 (8)
Asthenia	2 (4)

**Table 4:** Tumor types and best response to treatment. PR: partial response; SD: stable disease; PD: progressive disease; NA: not assessable.

Tumor types	Number of patients				
	Total	PR	SD	PD	NA
Nonsmall-cell lung	21	5	7	3	6
Small-cell lung	10	4	3	2	1
Gastrointestinal	10	3	3	2	2
Head and neck	3	0	1	2	0
Miscellaneous	6	1	3	1	1

Total	50	13	17	10	10

**Table 5:** Comparison of features of population based versus modified-Fibonacci method in phase I trials.

Features	Population based	Fibonacci
Number of patients required	~50	~20
Enrollment of patients	Continuous	Cohorts of three
Waiting time between cohorts	None	Required
Efficacy optimized	Yes	No
Preliminary efficacy data	Yes	Yes/No
